# Evidence Regarding Vitamin D and Risk of COVID-19 and Its Severity

**DOI:** 10.3390/nu12113361

**Published:** 2020-10-31

**Authors:** Joseph Mercola, William B. Grant, Carol L. Wagner

**Affiliations:** 1 Natural Health Partners, LLC, 125 SW 3rd Place, Cape Coral, FL 33991, USA; 2 Sunlight, Nutrition, and Health Research Center, P.O. Box 641603, San Francisco, CA 94164-1603, USA; wbgrant@infionline.net; 3 Department of Pediatrics, Shawn Jenkins Children’s Hospital, Medical University of South Carolina, 10 McClennan Banks Drive, MSC 915, Charleston, SC 29425, USA; wagnercl@musc.edu

**Keywords:** cathelicidin, COVID-19, endothelial dysfunction, IL-6, immune system, inflammation, MMP-9, SARS-CoV-2, vitamin D, 25-hydroxyvitamin D

## Abstract

Vitamin D deficiency co-exists in patients with COVID-19. At this time, dark skin color, increased age, the presence of pre-existing illnesses and vitamin D deficiency are features of severe COVID disease. Of these, only vitamin D deficiency is modifiable. Through its interactions with a multitude of cells, vitamin D may have several ways to reduce the risk of acute respiratory tract infections and COVID-19: reducing the survival and replication of viruses, reducing risk of inflammatory cytokine production, increasing angiotensin-converting enzyme 2 concentrations, and maintaining endothelial integrity. Fourteen observational studies offer evidence that serum 25-hydroxyvitamin D concentrations are inversely correlated with the incidence or severity of COVID-19. The evidence to date generally satisfies Hill’s criteria for causality in a biological system, namely, strength of association, consistency, temporality, biological gradient, plausibility (e.g., mechanisms), and coherence, although experimental verification is lacking. Thus, the evidence seems strong enough that people and physicians can use or recommend vitamin D supplements to prevent or treat COVID-19 in light of their safety and wide therapeutic window. In view of public health policy, however, results of large-scale vitamin D randomized controlled trials are required and are currently in progress.

## 1. Introduction

Until the 21st century, vitamin D was primarily recognized for its role in regulating calcium and bone health and preventing rickets [1]. In the last 20 years, however, research has shown that vitamin D also profoundly influences immune cells and generally lowers inflammation [2,3]. Vitamin D is a powerful epigenetic regulator, influencing more than 2500 genes [4] and impacting dozens of our most serious health challenges, including cancer [5,6], diabetes mellitus [7], acute respiratory tract infections [8], and autoimmune diseases such as multiple sclerosis [9].

According to the Worldometer website [10], the world had recorded 40,628,492 cases and 1,122,733 deaths from COVID-19 by 19 October 2020.

There are a number of findings regarding COVID-19 that may be related to vitamin D status.

- Seasonal dependence: it began in winter in the northern hemisphere and both case and death rates were lowest in summer, especially in Europe, and rates began increasing again in July, August, or September in various European countries [10]; it is thus generally inversely correlated with solar UVB doses and vitamin D production [11,12].
- African Americans and Hispanics have higher COVID-19 case and death rates than European Americans [13,14], possibly due to darker skin pigmentation and lower 25-hydroxyvitamin D [25(OH)D] concentrations [15].
- Much of the damage from COVID-19 is thought to be related to the “cytokine storm”, which is manifested as hyperinflammation and tissue damage [16].
- The body’s immune system becomes dysregulated in severe COVID-19 [17].

This narrative review examines the evidence indicating that vitamin D could play important roles in reducing the risk and severity of and death from infections, including COVID-19.

## 2. Findings Regarding Vitamin D and COVID-19

### 2.1. Vitamin D Deficiency Increases the Risk and Severity of COVID-19

Mainly owing to the recency and novelty of the SARS-CoV-2 virus, the evidence that vitamin D status affects the risk of COVID-19 comes primarily from observational and ecological studies. Clinical trials involving vitamin D supplementation and incidence of COVID-19 have not been reported to date. Of the 48 clinical trials on vitamin D and COVID-19 listed in the Clinical Trials registry maintained by the U.S. government [18], only four will investigate prevention, and three of those are enrolling health care workers, a group that is highly exposed to COVID-19.

Table 1 lists the findings from observational studies regarding 25(OH)D concentration and COVID-19 as of 15 October 2020, listed in ascending order by date first posted online. The table lists the study parameters and findings as well as the strengths and limitations of the studies. Two of the studies used 25(OH)D concentrations 10–14 years before the COVID-19 incidence data; the others generally used 25(OH)D concentrations at the time of hospital admission. Many of the studies have small numbers of COVID-19 patients. Other than the two studies with long intervals between 25(OH)D concentrations and COVID-19, and one observational study from Austria, the studies found inverse correlations between COVID-19 severity and/or risk of death.

The study from Newcastle upon Tyne, UK, supplemented patients with vitamin D_3_ depending on their baseline 25(OH)D concentration [24]. Those with 25(OH)D concentration <5 ng/mL were given 300,000 IU vitamin D_3_ followed by 1600 IU/d. Those with 25(OH)D between 5 and 10 ng/mL were given 200,000 IU vitamin D followed by 800 IU/d. Those with 25(OH)D between 10 and 16 ng/mL were given 100,000 IU vitamin D followed by 800 IU/d. Those with 25(OH)D between 16–30 ng/mL were given 800 IU/d, while those with 25(OH)D >30 ng/mL were not given vitamin D. Probably as a result, baseline 25(OH)D concentrations were not associated with mortality (*p* = 0.94).

Table 2 presents data on SARS-CoV-2 positivity for large populations independent of whether the participants had symptomatic COVID-19.

The study from Israel reported that 25(OH)D concentration inversely correlated with COVID-19 in both univariate and multivariate regression analyses except for multivariate hospitalization of patients [36]. For hospitalization of patients, the only significant factor in the multivariate hospitalization was age 50 years and older, implying that vitamin D status becomes less important with age. Yet, the study from the UK with patients of mean age 80 ± 10 years reported that 25(OH)D concentration was significantly lower for COVID-19 PCR+ patients than COVID-19 PCR– patients [29].

The observational study from the U.S. based on test data from Quest Diagnostics (Secaucus, NJ, USA) [38] is the largest observational study to date, with data for 191,779 patients with a mean age of 50 years (interquartile range, 40–65 years) tested for SARS-CoV-2 between 9 March and 19 June with 25(OH)D tests in the preceding 12 months at Quest Diagnostics. The study reported the following rates of SARS-CoV-2 positivity vs. 25(OH)D concentration: 39,120 patients <20 ng/mL, 12.5% (95% CI, 12.2–12.8%); 27,870 patients, 30–34 ng/mL, 8.1% (7.8–8.4%); 12,321 patients, >55 ng/mL, 5.9% (5.5–6.4%).

The finding that the SARS-CoV-2-positive rate in the U.S. varied from 6.5% for 25(OH)D concentration between 40 and 50 ng/mL to approximately 11.3% for 25(OH)D = 20 ng/mL may be due to the effect of vitamin D in reducing survival and replication of the virus by induction of cathelicidin and defensins as well as by increasing concentrations of free ACE2 [39], thereby preventing SARS-CoV-2 from entering cells via the ACE2 receptor [39]. The regression fit to all the data indicates that SARS-CoV-2 positivity is 40% lower for 25(OH)D >50 ng/mL than for 20 ng/mL, the value recommended by the Institute of Medicine [40,41]. The SARS-CoV-2-adjusted OR (aOR) for northern (>40°) vs. southern (<32°) was 2.66 (95% CI, 2.54–2.79), whereas that for central (32°–40°) vs. southern was 1.22 (1.16–1.38).

Regarding the higher rates in the northern states, a genetic variation was evident in SARS-CoV-2 from the original spike protein amino acid D614 form in China to the D614G mutated form it took in Europe [42]. (The Spike D614GF amino acid change is caused by an A-to-G nucleotide mutation at position 23,403 in the Wuhan reference strain.) The D614G form has greater transmission and was introduced to New York by people returning from Europe. Thus, that genetic change probably accounts for some of the higher SARS-CoV-2 positivity rate in the north. However, the shape of the 25(OH)D positivity rate is similar for all three latitude regions.

As for race/ethnic differences, African Americans have increased rates of social determinants predisposing them to COVID-19, such as lower income, education, and employment as well as higher rates of existing conditions such as diabetes, hypertension, cardiovascular disease, obesity, and lung disease [43]. Those factors may help explain why black people and Hispanic people have 7% and 4% higher SARS-CoV-2 positivity rates, respectively, than white people at 30 ng/mL. Nonetheless, the SARS-CoV-2 positive rate spread was much higher for black and Hispanic people than for white people near 20 ng/mL (18%, 16%, and 9%, respectively) than near 60 ng/mL (11%, 9%, and 5%, respectively), suggesting that vitamin D status plays a role in the increased COVID-19 rate for black and Hispanic people.

It can be argued that the association of low serum 25(OH)D concentrations with various diseases is due to “reverse causation”, i.e., that the disease state lowers the concentrations in proportion to the severity of the disease. That argument was made to explain why randomized controlled trials (RCTs) with vitamin D supplementation often fail to support observational studies reporting inverse correlations between 25(OH)D concentration and disease risk [44,45]. There are several counters to that argument.

One is that many vitamin D RCTs did not enroll participants with low 25(OH)D concentrations and did not supplement with sufficient vitamin D to produce a significant change in health outcome. Robert Heaney pointed out that vitamin D RCTs should be guided by serum 25(OH)D concentrations, not vitamin D dose [46] (see also, [47]). In addition, more recent RCTs have found that vitamin D supplementation can reduce risk of some of the non-skeletal health disorders considered by Autier in 2017: cancer incidence and death according to secondary analyses [48], cancer mortality rate [49] and progression from prediabetes to diabetes in the secondary analyses [7].

A second argument is that the 25(OH)D concentrations used in prospective observational studies are obtained from blood drawn prior to the disease outcomes of interest. Only three observational studies listed in Table 1 were prospective studies with less than one year lag between blood draw and COVID-19 or SARS-CoV-2 positivity [36,37,38].

A counter argument is that there is evidence that an acute-inflammatory disease state lowers 25(OH)D concentrations. A systematic review summarized results from eight studies reported between 1992 and 2013 regarding changes in 25(OH)D concentrations after acute inflammatory insult [50]. Four studies involved surgery. One involving 19 patients undergoing cardiopulmonary bypass reported an 8 ng/mL drop in five minutes with return to near baseline after 24 h [51]. Three involving knee or knee/hip arthroplasty or orthopedic surgery reported two-day decreases of 7 ng/mL [52], 4 ng/mL [53] and 1 ng/mL for males, 3 ng/mL for females [54]. There was no significant change for malarial infection [55] and a one ng/mL decrease for acute pancreatitis [56]. The largest decease was 15 ng/mL after three days for an injection of bisphosphonate [57]. The nearest outcome to COVID-19 was malaria infection, for which no change was found. Thus, from these studies, it is unclear whether acute inflammation not associated with surgery results in reduction in 25(OH)D.

### 2.2. Vitamin D and Treatment of COVID-10

A study by Ohaegbulam and colleagues involved four COVID-19^+^ patients in New York [58]. Two, a male aged 41 years and a female aged 57 years, were given five daily 50,000 IU vitamin D_2_ doses, whereas another two, males aged 53 and 74 years, were given five daily 1000 IU vitamin D_3_ doses. Baseline 25(OH)D concentration was between 17 and 22 ng/mL, whereas achieved 25(OH)D was 40 and 51 ng/mL for patients treated with high-dose vitamin D and 19 and 20 ng/mL for those treated with standard-dose vitamin D.

Biomarkers of inflammation were significantly reduced with high-dose treatment: CRP went from 31 to 2 mg/dL and from 17 to 8 mg/dL, compared with 13 to 22 mg/dL and 21 to 18 mg/dL for low-dose treatment; IL-6 went from 14 and 10 pg/mL to <5 pg/mL for high-dose treatment and from <5 and 6 pg/mL to <5 and 11 pg/mL for low-dose treatment.

The length of stay was 10 days for the high-dose patients and 13 and 14 days for the low-dose patients. The oxygen requirement went from zero and 15 L to zero for the high-dose patients and from 2 and 3 L to 2 and 7 L for the low-dose patients. The strengths of this study include that high-dose vitamin D_3_ supplementation was used and that baseline and post-supplementation values for many parameters were measured. The main limitation was that only two patients were supplemented with high-dose vitamin D_3_.

The results of pilot RCT of treatment of COVID-19 patients in Spain with calcifediol were announced on August 29 [59]. (Calcifediol [25(OH)D] is often used in Spain. It raises serum 25(OH)D concentration more quickly but does not last as long in the serum as a result of its lower lipophilia [60].) The mean age of the patients was 53 ± 10 years. None of the prognostic factors evaluated except previous high blood pressure [15 (58%) without treatment vs. 11 (24%) with treatment] significantly affected the outcome. In this study, 50 patients were given soft capsules of 0.532 mg of calcifediol on the day of admission, then 0.266 ng on day 3 and 7, and then weekly until discharge or admission to the intensive care unit (ICU). Thus, those in the treatment arm received approximately 130,000 IU of vitamin D during the first week, then approximately 33,000 IU/week thereafter. Serum 25(OH)D concentrations were not measured, but the calcifediol dose in the treatment arm was high enough to raise 25(OH)D concentration by approximately 20 ng/mL.

Forty-nine of the calcifediol-treated patients did not require the ICU, whereas 13 of the 26 not receiving that treatment did require the ICU. In addition, two of the patients admitted to the ICU died. The odds ratio (OR) for ICU in treated vs. control patients was 0.02 (95% CI, 0.002 to 0.17), which increased slightly when adjusted for hypertension and type 2 diabetes mellitus [OR = 0.03 (95% CI, 0.003 to 0.25)]. A meta-analysis of 34 studies found that hypertension was a significant risk factor for several or fatal COVID-19 compared to non-severe/non-fatal COVID-19: OR = 3.2 (95% CI 2.5 to 4.1) [61]. Thus, prevalence of hypertension should have been considered when dividing patients into treatment and control groups. The results of this study cannot be used for policy decisions. The main value of this study is that it is a pilot study for a study involving 1000 COVID-19 patients.

A “quasi-experimental study” of bolus vitamin D supplementation of residents in a French nursing home was conducted preceding and during a COVID-19 outbreak in the nursing home [62]. Residents were normally given a bolus dose of 80,000 IU vitamin D_3_ every two to three months. COVID-19 affected many of the residents starting in March 2020.

Fifty seven of the residents, who had received 80,000 IU vitamin D_3_ in the preceding month, were included in the “intervention group” while nine who had not were included in the “comparator group”. The mean age of the residents was 87 ± 9 years. The mean follow-up time was 36 ± 7 days. Forty-seven (83%) of the intervention group survived compared to only four (44%) of the comparator group (*p* = 0.02). The fully adjusted HR for mortality according to vitamin D supplementation was 0.11 (95% CI, 0.03 to 0.48, *p* = 0.003).

A clinical trial was conducted regarding bolus vitamin D dose (100,000 IU vitamin D_3_) supplementation involving 30 older (71 ± 6 years) and ten younger (38 ± 8 years) subjects and ten older controls (71 ± 10 years) [63]. Baseline 25(OH)D was 27 ± 8 ng/mL, rising to 42 ± 9 ng/mL within six days, then falling in a linear fashion to 32 ng/mL after 70 days. Thus, bolus vitamin D_3_ supplementation monthly would be appropriate for nursing-home residents.

### 2.3. Vitamin D Helps Immune Cells Produce Antimicrobial Peptides

**Figure 1:**
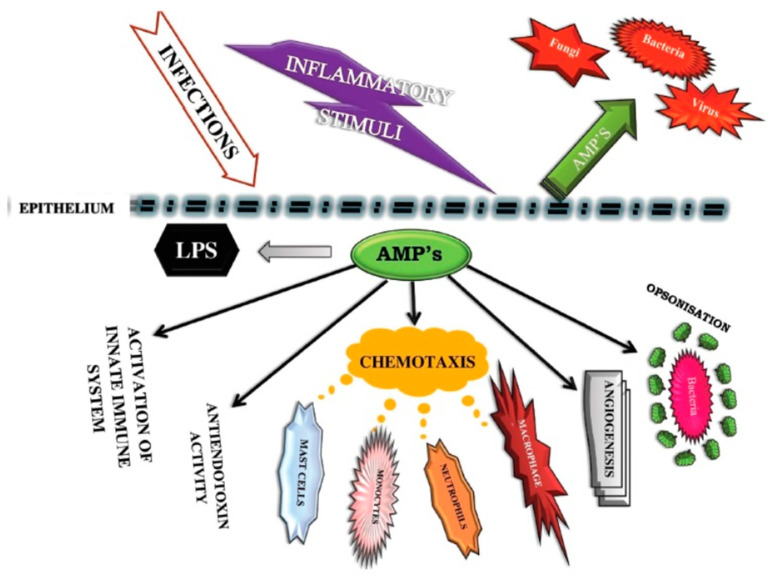
The cascade of events by the innate immune system in response to viral infections. Among the functions of AMPs (antimicrobial peptides) is chemotaxis, the movement of cells in response to a chemical stimulus, here macrophages, mast cells, monocytes, and neutrophils. Other effects include activation of the innate immune system, effects on angiogenesis, antiendotoxin activity, and opsonization (the molecular mechanism whereby pathogenic molecules, microbes, or apoptotic cells (antigenic substances) are connected to antibodies, complement, or other proteins to attach to the cell surface receptors on phagocytes and NK cells). LMS (lipopolysaccharide)

Many studies have shown that vitamin D activates immune cells to produce AMPs, which include molecules known as cathelicidins and defensins [64,65,66,67]. AMPs have a broad spectrum of activity, not only antimicrobial but also antiviral, and can inactivate the influenza virus [68]. The antiviral effects of AMPs are the result of, among other effects, the destruction of envelope proteins by cathelicidin [69,70,71]. See Figure 1.

Cathelicidins are a distinct class of proteins present in innate immunity of mammals. In humans, the primary form of cathelicidin is known as LL-37 [72]. LL-37 also blocks viral entry into the cell similarly to what is seen with other antimicrobial peptides [73].

### 2.4. Vitamin D Reduces Inflammatory Cytokine Production

Elevated inflammation is an important risk factor for COVID-19 [16]. For example, much of the pathogenesis surrounding COVID-19 infection involves microvascular injury induced by hypercytokinemia, namely, by an important inflammatory cytokine—interleukin 6 (IL-6) [74,75]. Thus, it is useful to examine the role of vitamin D in reducing inflammation.

A number of reviews have suggested that one of the hallmarks of COVID-19 severity is the presence of a “cytokine storm” [76,77,78,79]. The “cytokine storm” is defined as the state of out-of-control release of a variety of inflammatory cytokines [79]. Observational studies, however, have found that cytokine concentrations are elevated in COVID-19 patients compared to controls, but not as high as in some other diseases.

A study in the Netherlands compared cytokine levels in critically ill patients [80]. The study involved 46 COVID-19 patients, 51 with septic shock with acute respiratory tract syndrome (ARDS), 15 with septic shock without ARDS, 30 with out-of-hospital cardiac arrest (OHCA), and 62 with trauma. Levels of (TNF_α_) for COVID-19 patients were lower than for septic shock patients but higher than for OHCA or trauma patients. Levels of IL-6 and IL-8 for COVID-19 patients were lower than for septic shock patients but comparable with those for OHCA and trauma patients.

A recent review examined whether IL-6 concentrations might affect the outcome of COVID-19 [75]. The evidence presented included age-stratified IL-6 concentrations from a healthy Italian population were highly correlated with age-stratified Italian COVID-19 deaths, which in turn were highly correlated with age-stratified COVID-19 death rates in the UK. The researchers also cited trials of vitamin D supplementation and its effect on IL-6 concentrations, of which eight of 11 showed a significant lowering of IL-6. People for whom a significant lowering was not found were healthy older adults, asthma patients, and prediabetic adults. That reviewshowed how IL-6 increases the severity of COVID-19 by upregulating angiotensin-converting enzyme 2 (ACE2) receptors and induction of macrophage cathepsin L. Cathepsin L mediates the cleavage of the S1 subunit of the coronavirus surface spike glycoprotein. That cleavage is necessary for coronavirus entry into human host cells, virus–host cell endosome membrane fusion, and viral RNA release for the next round of replication [81].

A study from Ireland investigated cytokine concentrations of healthy controls, stable COVID-19 patients, ICU COVID-19 patients, and ICU community-acquired pneumonia patients [75]. ICU-COVID-19 patients had the highest concentrations of IL-1β, IL-6, IL-6 to IL-10 ratio, and tumor necrosis factor receptor superfamily member 1A (TNFR1). Stable COVID-19 patients had concentrations that were between the levels noted for healthy controls and those of ICU COVID-19 patients for all of the cytokines. ICU-community-acquired pneumonia patients had inflammatory cytokine concentrations between stable and ICU COVID-19 patients but higher IL-10 concentrations.

A study of COVID-19 hyperinflammation (COV-HI) was conducted on 269 polymerase chain reaction (PCR)-confirmed COVID-19 patients admitted to two UK hospitals in March [82]. Hyperinflammation was defined as CRP concentration greater than 150 mg/L or doubling within 24 h from greater than 50 mg/L, or a ferritin concentration greater than 1500 µg/L. Ninety (33%) of the patients met the criteria for COV-HI at admission. Forty percent of COV-HI patients died compared to 26% of the non-COV-HI patients. Meeting the COV-HI criteria was significantly associated with risk of next-day escalation of respiratory support or death (hazard ratio = 2.24 (95% CI, 1.62 to 2.87)).

Another study developed a more extensive set of criteria for COV-HI [83]. The criteria included elevated temperature, macrophage activation (elevated ferritin), haemotological dysfunction related to neutrophils and lymphocytes, coagulopathy (elevated D-dimer), hepatic injury (elevated lactate dehydrogenase or aspartate aminotransferase concentration), and cytokinaemia (elevated IL-6, triglyceride, or CRP concentration). It is not clear whether vitamin D supplementation could affect any of these factors other than cytokinaemia.

Other papers have noted that concentrations of many cytokines are elevated in COVID-19 patients [75,84,85].

There are several reasons why the cytokine storm is associated with severe COVID-19 and death [86,87]. As outlined in the review by Hojyo [86], the hypothesis that the main cause of death of COVID-19 is ARDS with cytokine storms can be explained by at least two reasons. One is intravascular coagulation as an important cause of multiorgan injury, which is mainly mediated by inflammatory cytokines such as IL-6 [88]. The other is that the SARS-CoV-2 virus affects endothelial cells, causing further cell death, which leads to vascular leakage and induces a cytopathic effect on airway epithelial cells [89].

### 2.5. Type II Pneumocytes and Surfactants in the Lungs

The type II pneumocytes in the lung are the primary target for coronaviruses because the ACE2 receptors to which the virus binds are highly expressed on those cells. One problem with COVID-19 is that it impairs the function of type II pneumocytes, which then decreases the surfactant concentration in the alveolar–air interface [90]. That is important because surfactant prevents the collapse of the alveoli.

Surfactant allows alveoli to stay open and compliant during both inhalation and exhalation. During inhalation, alveoli may collapse if they do not contain surfactant. If they collapse, gas exchange across the alveoli wall cannot occur. Without surfactant, each breath taken is like blowing up a collapsed balloon and then letting the air out of that balloon (lungs) and then doing it all over again with the next breath cycle. Simply put, having enough surfactant is necessary for alveoli to stay open and gas exchange to occur. Another aspect of surfactant is its protein A (SP-A), which binds to influenza A viruses via its sialic acid residues and thereby neutralizes the virus [91]. Surfactant protein D clears influenza A from the lungs of mice [92]. There is some evidence that 1α,25(OH)_2_D increases surfactant production [93]. Such activity can be generalized to other viruses.

### 2.6. Vitamin D, Angiotensin II, and ACE2 Receptors

Angiotensin-converting enzyme (ACE) is part of the renin–angiotensin system (RAS), which controls blood pressure by regulating the volume of bodily fluids. Angiotensin-converting enzyme 1 (ACE1) converts the hormone angiotensin I to the active vasoconstrictor angiotensin II [94]. Angiotensin II is a natural peptide hormone best known for increasing blood pressure through stimulating aldosterone [95] ACE2 normally consumes angiotensin I, thereby lowering its concentrations. However, SARS-CoV-2 infection downregulates ACE2, leading to excessive accumulation of angiotensin II.

Cell cultures of human alveolar type II cells with vitamin D have shown that the SARS-CoV-2 virus interacts with the ACE2 receptor expressed on the surface of lung epithelial cells. Once the virus binds to the ACE2 receptor, it reduces its activity and, in turn, promotes ACE1 activity, forming more angiotensin II, which increases the severity of COVID-19 [96,97]. That effect may also be related to the vitamin D binding protein [98].

The vitamin D metabolite calcitriol increases expression of ACE2 in the lungs of experimental animals [99]. (Calcitriol has also been found to increase ACE2 protein expression in rat microglia BV2 cells [100].) The additional ACE2 expressed as a consequence of vitamin D supplementation might reduce lung injury [101] because it can promote binding of the virus to the pulmonary epithelium. As mentioned, calcitriol also induces α-1-antitrypsin synthesis, which is vital for lung integrity and repair, by CD4^+^ T cells, which is required for the increased production of anti-inflammatory IL-10. Calcitriol should not be used to treat COVID-19 given the risk of hypercalcemia; however, vitamin D supplementation increases calcitriol concentrations [102] through its regulated conversion in the proximal tubules of the kidney and in extrarenal cells at the nuclear membrane.

High concentrations of angiotensin II may cause ARDS or cardiopulmonary injury. Renin, by contrast, is a proteolytic enzyme and a positive regulator of angiotensin II. Vitamin D is a potent inhibitor of renin. Vitamin D supplementation prevents angiotensin II accumulation and decreases proinflammatory activity of angiotensin II by suppressing the release of renin in patients infected with COVID, thus reducing the risk of ARDS, myocarditis, or cardiac injury [103].

**Figure 2:**
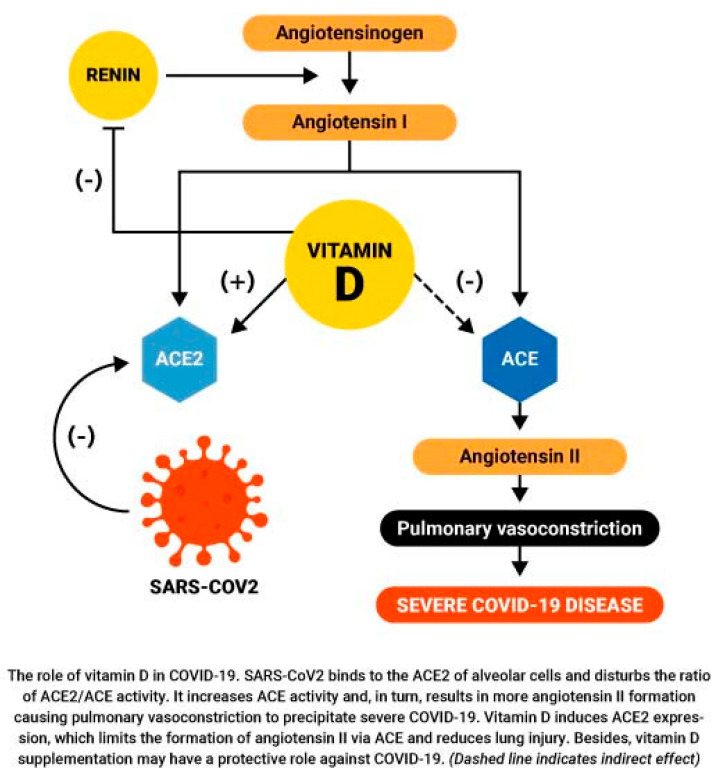
The role of vitamin D regarding ACE in response to SARS-CoV-2. ACE: angiotensin-converting enzyme.

Although vitamin D increases expression of ACE2, which promotes the binding of the virus, it prevents the constriction response of the lung blood vessel in COVID-19, as illustrated in Figure 2 [104] (permission to reuse granted by copyright holder). ARDS is also due to a variety of mechanisms, including cytokine storm, neutrophil activation, and increased (micro)coagulation, and it is likely that vitamin D supplementation would counter those mechanisms [105]. ARDS is responsible for approximately 70% of fatal COVID-19 cases [106].

### 2.7. Reduces Risk of Endothelial Dysfunction

Jun Zhang and colleagues outlined how endothelial dysfunction (ED) contributes to COVID-19-associated vascular inflammation and coagulopathy, two hallmarks of severe COVID-19 [107]. Four stages of ED were identified that contribute to inflammation and coagulopathy. Stage 1 is Type I endothelial cell (EC) activation after infection by SARS-CoV-2 entering through the ACE2 receptor. That results in the loss of anticoagulant molecules. Stage 2 is Type II EC activation which leads to the de novo synthesis of procoagulant molecules. Stage 3 is endothelial apoptosis involving endothelial detachment and denudation of basement membrane. Stage 4 is endothelial necrosis.

A number of papers have discussed how vitamin D can reduce risk of ED. In a second review, Zhang and colleagues notes that vitamin D likely protects against ED by reducing oxidative stress and NF-κB activation [108,109]. A recent review outlined how vitamin D maintains endothelial function by reducing the production of reactive oxygen species as well as reducing proinflammatory mediators such as TNF-α and IL-6 and suppressing the NF-κB pathway [110]. A laboratory study involving mice as well as type II alveolar epithelial cells found that vitamin D attenuated lung injury by stimulating type II alveolar epithelial cell proliferation and migration, reducing epithelial cell apoptosis and inhibiting TGF-β-induced epithelial mesenchymal transition [111].

### 2.8. Matrix Metalloproteinase 9

Matrix metalloproteinase-9 (MMP-9) is a member of the family of proteases that degrade extracellular matrix remodeling proteins. MMP-9 has been widely studied in acute lung injury and acute lung disease [112]. A study in Norway investigated correlations between respiratory failure and MMP-9 in 21 COVID-19 patients with respiratory failure in comparison with seven COVID-19 patients without respiratory failure [113].

Respiratory failure was defined as arterial partial pressure of oxygen to fraction of inspired oxygen ratio (P/F ratio) <40 kPa (300 mmHg), corresponding to the threshold in ARDS. The researchers found a significant inverse correlation of the P/F ratio with respect to the log_10_ (MMP-9) as well as significantly higher MMP-9 concentrations for P/F below the threshold than above it. In a study of 171 healthy British Bangladeshi adults, vitamin D status was the sole determinant of circulating MMP-9 (inversely) and an independent determinant of CRP (inversely) [114].

A search of pubmed.gov for articles regarding vitamin D, MMP-9 and infections did not find any related to viral infections, but did find some regarding bacterial infections. A laboratory study in the UK found that *Mycobacterium tuberculosis* induced the production of MMP-1, MMP-7, and MMP-10 [115]. MMP-9 gene expression, secretion and activity were significantly inhibited by 1α,25(OH)_2_D_3_ irrespective of infection.

### 2.9. RAS-Mediated Bradykinin Storm

Several recent publications looked at the role of bradykinin (BK) in the progression of COVID-19. Jacobson used the Summit supercomputer at Oak Ridge National Lab in Tennessee is the second fastest supercomputer in the world and in the summer of 2020 analyzed data on more than 40,000 genes from 17,000 samples from COVID-19 patients [116]. The analysis revealed that SARS-CoV-2 actively upregulates ACE2 receptors in places where they’re typically expressed at low levels, including the lungs.

Additionally, an imbalance in RAS was also found, represented by decreased ACE in combination with increases in ACE2, renin, angiotensin, key RAS receptors, kininogen and many kallikrein enzymes that activate it, and both BK receptors, which produces a BK storm [116]. Since BK dilates blood vessels and increases permeability, excessive BK leads to fluid to soft tissue fluid accumulation. This leads to several adverse effects seen in COVID-19 patients, including on the heart, vascular system, pulmonary system, brain, and muscles [116]. The authors suggested that vitamin D could reduce the risk of the BK storm through several mechanisms including regulation of RAS.

Renin is the enzyme that catalyzes the first step in the activation pathway of angiotensinogen by cleaving angiotensinogen to form angiotensin I, which is then converted to angiotensin II by angiotensin I converting enzyme. In the COVID samples Jacobson analyzed renin levels were increased 380-fold compared to controls. Vitamin D is a negative endocrine RAS modulator and inhibits renin expression and generation [40] and it appears likely that vitamin D deficiency amelioration would limit the COVID-19 BK storm. However, further investigation is needed to evaluate the role of vitamin D in this context

### 2.10. Summary: How Vitamin D Might Reduce Risk, Severity, and Death from COVID-19

Many reviews consider the ways in which vitamin D reduces the risk of viral infections [8,15,76,117,118,119,120,121,122,123,124,125]. Vitamin D probably reduces the risk of viral respiratory infections because it influences several immune pathways [126].

Vitamin D appears to decrease the risk of respiratory tract infections, including COVID-19, through six potential mechanisms:

- Inactivates some viruses by stimulating antiviral mechanisms such as antimicrobial peptides, as discussed in Section 2.3.
- Reduces proinflammatory cytokines through modulating the immune system, as discussed in Section 2.4.
- Increases ACE2 concentrations and reduces risk of death from ensuing ARDS, as discussed in Section 2.5.
- Reduces risk of endothelial dysfunction, as discussed in Section 2.7.
- Reduces MMP-9 concentrations, as discussed in Section 2.8.
- Reduces risk of the bradykinin storm, as discussed in Section 2.9.

However, much further research is required to confirm the mechanisms by which vitamin D reduces the risk and severity of COVID-19.

### 2.11. Vitamin D Seasonality and COVID-19

Since epidemic influenza rates are higher in winter than in summer [127], it was expected that COVID-19 would also exhibit a seasonal dependence. Two recent papers provide evidence on monthly and seasonal variation of viral infections. One in 2019 performed a systematic analysis of global patterns in monthly activity of influenza virus, respiratory syncytial virus, parainfluenza virus and metapneumovirus [128].

The second one, published in 2020, did the same for the global seasonality of human seasonal coronaviruses [129]. For nearly all of these viruses, infection rates in northern mid and high latitudes are highest from November through March. They examined correlations of meteorological conditions with coronavirus infections, finding the highest correlation with low temperature combined with high relative humidity. In winter, high relative humidity is associated with low absolute humidity. Low absolute humidity was found as an important factor for transmission of epidemic influenza [130].

A recent analysis of influenza seasonality in northern Europe found that low temperature was the most important factor facilitating transmission, followed by solar UV radiation and low humidity [131]. That paper also noted that high humidity favors transmission in tropical and subtropical zones, in accordance with the findings by Li et al. [129]. According to data posted at WorldoMeter [10], COVID-19 case rates in Northern Europe peaked in spring, were very low in summer, then started rising in July (e.g., Spain), August (e.g., Italy) or September (e.g., the UK).

At higher latitudes in the southern hemisphere, COVID-19 rates were very low through April, then started to rise in June and continued rising into October as in Argentina. On the other hand, in the tropical South American countries such as Brazil, COVID-19 rates started rising in April, peaking around early August then declined, in general agreement with other coronavirus infections [129]. Of course, a number of factors help determine the case rate including the extent to which social distancing and mask wearing are practiced, when school attendance begins, and solar and meteorological factors. However, mortality rates were only high in the spring. Most likely the low mortality rates in September were due to the COVID-19 rates being highest for those aged 20 to 29 years [132]. Yet, with time, COVID-19 rates will increase among the elderly as well.

### 2.12. Racial/Ethnic Disparities

As mentioned in the introduction, African American and Hispanic individuals have higher COVID-19 case and death rates than European Americans [13,14], possibly due to darker skin pigmentation and lower 25(OH)D concentrations [15]. Confounding these findings, however, is that both African Americans and Hispanics are also at greater risk of COVID-19 due to other factors such as working and living in close proximity to many people and having higher rates of hypertension and other chronic diseases such as type II diabetes, often associated with COVID-19 [133].

The findings regarding SARS-CoV-2 positivity by race/ethnicity from the Quest Diagnostics data set are useful regarding racial/ethnic variations in risk of COVID-19 [38]. Mean serum 25(OH)D concentrations for different racial/ethnic groups in the U.S. can be used to estimate the effect of vitamin D status on the risk of COVID-19. Figure 2 shows that Black non-Hispanics with 25(OH)D ≤20 ng/mL had a 19% SARS-CoV-2 positivity, Hispanics with 25(OH)D concentration = 21 ng/mL had 15% positivity, while white non-Hispanics with 25(OH)D concentrations near 26 ng/mL had a positivity near 8%. If black non-Hispanics had a mean 25(OH)D concentration near 26 ng/mL, it is projected that they would have a positivity of approximately 17%.

Thus, the contribution of vitamin D status to positivity higher than for white non-Hispanics is 2%(19%–8%) ~20%, while that for Hispanics is 2%(15%–8%) to ~30%. Thus, while disparities in vitamin D status do not explain much of the ethnic/racial differences in SARS-CoV-2 positivity, if black non-Hispanics were to raise their mean serum 25(OH)D concentration to 50 ng/mL, they could lower risk by approximately 40%, Hispanics by ~50%, and white non-Hispanics by ~25%. A recent letter suggested that African Americans have a high risk of severe disease and mortality by SARS-CoV-2 due to vitamin D deficiency [134]. The mechanism proposed was reduced ACE2 due to vitamin D deficiency.

An analysis of physician deaths in the UK showed that 18 of 19 doctors and dentists who died by 22 April 2020, were of black, Asian, and mixed ethnicity [135]. Presumably, they were not of low socioeconomic status and had similar contact with patients as their white counterparts. They could have had low vitamin D status due to darker skin and/or vegetarian or vegan diets. In England, a study involving white residents reported that vegans and vegetarians have 25(OH)D concentrations as much as 8 ng/mL lower than those of meat eaters [136].

### 2.13. Vitamin D Reduces Risk of COVID-19 in a Causal Manner

Hill’s criteria for causality offer a scientific approach to determine causal relationships in biological systems [137]. The important criteria for vitamin D include temporality, strength of association, dose–response relationship, consistency of findings, plausibility (e.g., mechanisms), accounting for alternate explanations, experiment (e.g., randomized controlled trial), and coherence with known facts.

Annweiler and colleagues evaluated the evidence that vitamin D reduces the risk and severity of COVID-19 in a causal manner [138]. An updated summary of the evidence is presented in Table 3. Most of the criteria are satisfied. A number of mechanisms have been identified or proposed regarding how vitamin D could reduce risk of COVID-19. Further experimental verification is warranted for some of them.

The pilot calcifediol treatment RCT conducted in Spain was of low quality due to the low number of participants and failure to measure 25(OH)D concentrations [59]. While the meta-analysis of acute respiratory tract infections found a significant reduction with respect to vitamin D supplementation in RCTs [8], vitamin D supplementation does not reduce risk of all respiratory tract infections, e.g., pneumonia in infancy and early childhood [140].

Hill stated: “None of my nine viewpoints can bring indisputable evidence for or against the cause-and-effect hypothesis and none can be required as a *sine qua non*. What they can do, with greater or less strength, is to help us to make up our minds on the fundamental question—is there any other way of explaining the set of facts before us, is there any other answer equally, or more, likely than cause and effect?” p. 36 in [137].

Evidence-based medicine (EBM) has generally come to mean a heavy reliance on RCTs. Yet, that was only one type of evidence proposed by Sackett, the father of EBM. The practice of evidence-based medicine means integrating individual clinical expertise with the best available external clinical evidence from systematic research. By best available external clinical evidence, we mean clinically relevant research, often from the basic sciences of medicine, but especially from patient-centered clinical research into the accuracy and precision of diagnostic tests (including the clinical examination), the power of prognostic markers, and the efficacy and safety of therapeutic, rehabilitative, and preventive regimens [141].

Indeed, several reviews of EBM have discussed the relative roles of RCTs and observational studies. A review from 2004 compared results from RCTs and observational studies for four health outcomes, reporting that if a reasonable number of each type of study was available, the results were very similar [142]. A review from 2010 proposed a hierarchy with meta-analysis on top, followed by systematic review, RCT, and so on [143].

A review tabulated the ways both RCTs and their meta-analyses could have biased results, either in domains or in design [144]. One design bias is the wrong dose, often a problem with vitamin D RCTs in that vitamin D doses have generally been 1000 IU/d or less until recently. Another problem is enrolling participants with relatively high 25(OH)D concentrations and giving doses too low to be effective [46]. Finally, a review published in 2017 compared RCTs with “real-world studies” (observational studies) [145]. Among other strengths, observational studies generally include more diverse and larger populations than RCTs.

Regarding the comparison of findings for vitamin D from observational studies and RCTs, they are in general agreement—though with some caveats. RCTs support the role of vitamin D supplementation in reducing the risk of acute respiratory tract infections (ARTIs) [8]. However, an RCT reporting that vitamin D supplementation reduced risk of influenza type A for schoolchildren showed no reduction for influenza type B [146]. Vitamin D_3_ supplementation (14,000 IU/wk) did not result in a lower risk of tuberculosis infection, tuberculosis disease, or ARTIs than placebo among vitamin D-deficient schoolchildren in Mongolia [147]. Thus, vitamin D supplementation does not reduce risk of all types of respiratory tract infections in all places.

### 2.14. Other Nutrients That May Augment the Effectiveness of Vitamin D Supplementation

Magnesium supplementation is recommended when taking vitamin D supplements. Magnesium facilitates vitamin D-related processes. All the enzymes that metabolize vitamin D seem to require magnesium, which acts as a cofactor in the enzymatic reactions in the liver and kidneys [148]. The dose of magnesium should be in the range of 250–500 mg/d. Magnesium activates more than 600 enzymes and influences extracellular calcium concentrations [149]. It is essential for the stability of cell function, RNA and DNA synthesis, and cell repair, as well as maintaining the cell’s antioxidant status. Magnesium is an important cofactor for activating a wide range of transporters and enzymes [150,151], many of which involve vitamin D metabolism.

Although vitamin D is likely to be the most important nutrient to optimize COVID-19 prevention, other nutrients, such as magnesium, vitamin K_2_ and other micronutrients, are also known to impact the immune system and infection risk [152,153,154].

## 3. Conclusions

As discussed here, there is emerging evidence that higher serum 25(OH)D concentrations are associated with the reduced risk and severity of COVID-19. It might do so through a variety of mechanisms, such as maintaining intact epithelial layers, reducing the survival and replication of viruses, reducing the production of pro-inflammatory cytokines, and increasing ACE2 concentrations. More research is required to evaluate the mechanisms whereby vitamin D might reduce the risk of COVID-19.

The strongest evidence to date comes from 14 observational studies that report inverse correlations between serum 25(OH)D concentrations and SARS-CoV-2 positivity and/or COVID-19 incidence, severity and/or death. Hill’s criteria for causality in a biological system are largely satisfied for vitamin D in reducing risk of COVID-19, with the exception of successful large-scale vitamin D supplementation RCTs demonstrating significantly reduced risk of or improved outcome for COVID-19. Such RCTs are now under way [18,155]. Until then, individuals and physicians can use vitamin D supplementation as they wish, but public health policies likely will not include vitamin D to reduce risk or death from COVID-19 until large-scale RCTs are reported demonstrating significant reductions in COVID-19 incidence, severity, and/or death from vitamin D supplementation.

## Figures and Tables

**Table 1:** Summary of observational study findings regarding COVID-19 and 25(OH)D concentrations posted at pubmed.gov by 27 September 2020.

	Location	Participants	Outcomes vs. 25(OH)D (ng/mL)	Strengths, Limitations	Reference
1	UK	449 C19 patients 348,598 controls from UK Biobank	Incidence for 25(OH)D <10 vs. >10 Univariable OR = 1.37 (1.07–1.76, *p* = 0.01) Multivariable OR = 0.92 (0.71–1.21, *p* = 0.56)	Some confounding variables should not be used since they affect 25(OH)D concentrations [19,20] 25(OH)D data were from blood drawn from 2006 to 2010 Participant 25(OH)D concentrations change over time, reducing correlations with disease outcomes [21]	Hastie [22]
2	Switzerland	27 patients PCR+ for SARS-CoV-2; 80 patients PCR– 1377 controls with 25(OH)D measured in same period in 2019	Patients PCR+ had mean 25(OH)D = 11 vs. 25 for patients PCR– (*p* = 0.004) Controls had 25(OH)D = 25, not significantly different from patients PCR– (*p* = 0.08)	PCR+ is for antibodies; may not be active COVID-19 Small number of PCR+	D’Avolio [23]
3	UK, Newcastle upon Tyne	92 C19, non-ITU; 42 C19, ITU Patients were supplemented with vitamin D_3_ at doses inversely correlated with baseline 25(OH)D concentration	Non-ITU vs. ITU: 25(OH)D 19 ± 15 vs. 13 ± 7 (*p* = 0.30) 25(OH)D <20 vs. >20 (*p* = 0.02) RR for death, 25(OH)D = 0.97 (0.42–2.23, *p* = 0.94)	Lack of correlation of death with baseline 25(OH)D was likely due to graded supplementation with vitamin D	Panagiotou [24]
5	Italy	42 C19 hospitalized patients; mean age 65 ± 13 years, 88 with ARDS	!L6 for 25(OH)D >30: 80 ± 40 pg/L; for 25(OH)D <10, 240 ± 470 pg/L After 10 days, patients with 25(OH)D <10 had a 50% mortality vs. 5% for 25(OH)D <10 (*p* = 0.02)	Patients with 25(OH)D <10 ng/mL had a mean age of 74 ± 11 years vs. 63 ± 15 years for patients with 25(OH)D ≥10 ng/mL	Carpagnano [25]
6	Korea	50 C19 patients with PCR+, 150 controls; mean age = 52 ± 20 years	C19 vs. control: 16 (SD 8) vs. 25 (SD 13) (*p* < 0.001); ≤20, 74% vs. 43% (*p* = 0.003); ≤10, 24% vs. 7% (*p* = 0.001)	Strengths: measured B vitamin, folate, selenium and zinc concentrations as well as 25(OH)D Weaknesses: small number of patients; incomplete analysis of data for C19 outcomes	Im [26]
7	Russia	80 C19 patients with community-acquired pneumonia	Severe: 25(OH)D = 12 ± 6 ng/mL; moderate to severe: 25(OH)D = 19 ± 14 ng/mL Death: 25(OH)D = 11 ± 6 ng/mL; discharged: 18 ± 6 ng/mL Obesity rates: 62% for severe, 15% for discharged, *p* < 0.001	Strengths: studied the effect of obesity Weaknesses: small numbers	Karonova [27]
8	Mexico	172 hospitalized C19 patients	Mean 25(OH)D = 17 ± 7 ng/mL for hospitalized C19 patients Survivors: mean age = 48 ± 13 years; 25(OH)D = 17 ± 7 ng/mL Death: mean age = 65 ± 12 years; 25(OH)D = 14 ± 6 ng/mL (*p* value for difference in 25(OH)D = 0.0008)	Weaknesses: survivors were much younger than non-survivors Comorbid factors not reported	Tort [28]
9	UK	105 patients with C19 symptoms; 70 C19 PCR+, 35 PCR–; mean age = 80 ± 10 years	PCR+: 25(OH)D = 11 (8–19); PCR–: 25(OH)D = 21 (13–129) (*p* = 0.0008) Comorbid diseases were not significantly correlated with ≤12 vs. >12;	PCR+ is for antibodies; may not be active COVID-19	Baktash [29]
10	UK	656 C19, 203 died from C19; 340,824 controls from UK Biobank	Incidence for 25(OH)D <10 vs. >10 Univariable OR = 1.56 (1.28–1.90, *p* < 0.0001) Multivariable OR = 1.10 (0.88–1.37, *p* = 0.40) Death for 25(OH)D <10 ng/mL vs. >10 ng/mL Univariable OR = 1.61 (1.14–2.27, *p* = 0.0007) Multivariable OR = 1.21 (0.83–1.76, *p* = 0.31)	Same comments as for earlier UK Biobank study	Hastie [30]
11	Germany	185 C19; median age = 60 years	Multivariable HR for death for 25(OH)D <12: IMV/D, 6.1 (2.8–13.4, *p* < 0.001); D, 14.7 (4.2–52.2, *p* < 0.001)	Strengths: HR adjusted for age, gender, and comorbidities Weaknesses: Small number of IMV and deaths	Radujkovic [31]
12	Austria	109 C19 hospitalized patients; mean age = 58 ± 14 years	Mild: 26 ± 12 Moderate: 22 ± 8 Severe: 20 ± 10 (*p* = 0.12) PTH increased significantly with age (*p* = 0.001)	The vitamin D finding may have been limited owing to the high mean 25(OH)D concentrations Mild C19 patients had mean age = 46 ± 16 years; moderate and severe patients has mean age = 60 ± 13 years PTH increases with age [32]	Pizzini [33]
13	Spain	80 emergency department patients with a PCR+ test within the past three months; retrospective study	49 non-severe C19, 25(OH)D = 19 ng/mL; 31 severe C19, 25(OH)D = 13 ng/mL (*p* = 0.15) For patients under 65 years, 30 non-severe C19, 25(OH)D = 22 (11–31) ng/mL; 10 severe C19, 25(OH)D = 11 (9–12) ng/mL (*p* = 0.009) Multivariable OR for severe C19 for 25(OH)D <20 ng/mL = 3.2 (95% CI, 0.9 to 11.4, *p* = 0.07)	Weaknesses: small study; prevalence of advanced chronic kidney disease was higher in severe than non-severe cases (45% vs. 24%, *p* = 0.054)	Macaya [34]
14	China	62 C19 patients, 80 healthy controls	age, 25(OH)D: controls: 43 years, 29 (23–33) ng/mL; mild/moderate C19: 39 (30–49) years, 23 (18–27) ng/mL; severe/critical C19: 65 (54–69) years, 15 (13–20) ng/mL Multivariate OR for severe/critical C19 for 25(OH)D <20 ng/mL = 15 (1.2 to 187, *p* = 0.03)	Strengths: many factors measured Weaknesses: the severe/critical patients were much older than mild/moderate patients and controls	Ye [35]

Abbreviations: ARDS, acute respiratory distress syndrome; C19, COVID-19 patients; D, death; HR, hazard ratio; IMV, invasive mechanical ventilation; ITU, intensive treatment unit; OR, odds ratio; PCR, polymerase chain reaction; PTH, parathyroid hormone; RR, relative risk; SD, standard deviation.

**Table 2:** Summary of observational study findings regarding SARS-CoV-2 positivity in general populations and 25(OH)D concentrations by date of first publication up to October 15, 2020.

	Location	Participants	Outcomes vs. 25(OH)D (ng/mL)	Strengths, Limitations	Reference
1	Israel	Data from a hospital in Tel Aviv involving patients who had previous 25(OH)D measurements and were tested for SARS-CoV-2 using PCR 782 patients PCR+ 7025 patients PCR–	Univariate: 20–29 vs. >30: OR = 1.59 (1.24–2.02, *p* = 0.005); <20 vs. >30, OR = 1.58 (1.13–2.09, *p* = 0.0002). Multivariate: <30 vs. >30, OR = 1.50 (1.13–1.98, *p* = 0.001)	Strengths: large number of participants. Weakness: PCR+ is not COVID-19.	Merzon [36]
2	US	489 C19 patients, PCR+; mean age = 49 ± 18 years with 25(OH)D concentrations were from preceding 12 months	124 <20 vs. 287 >20, RR = 1.77 (1.12–2.81, *p* = 0.02)	Strengths: this is a retrospective study in which serum 25(OH)D concentrations and vitamin D supplementation history were obtained during the preceding 12 months.	Meltzer [37]
3	US	191,779 patients tested for 25(OH)D and SARS-CoV-2 positivity during the past year by Quest Diagnostics	SARS-CoV-2 positivity for 25(OH)D <20 = 12.5% (95% CI, 12.2–12.8%); positivity for 25(OH)D >55 = 5.9% (95% CI, 5.5–6.4%). For 25(OH)D <20, SARS-CoV-2 positivity rates were: black non-Hispanic, 19%; Hispanic, 16%; white non-Hispanic, 9%	Strengths: large number of participants and is a retrospective study. 25(OH)D concentrations were seasonally adjusted. Weaknesses: SARS-CoV-2 positivity is a precursor to COVID-19, but many with positivity do not develop COVID-19. There may be bias in who was tested since the tests were ordered by physicians.	Kaufman[38]

**Table 3:** Hill’s criteria for causality applied to vitamin D and COVID-19.

Criterion	Evidence	Reference
Strength of association	A retrospective study in Chicago found a 77% increased risk of COVID-19 for 25(OH)D <20 ng/mL vs. >20 ng/mL	[37]
Consistency	Thirteen of 16 observational studies of COVID-19 or SARS-CoV-2 positivity reported inverse correlations with respect to 25(OH)D concentration. Two studies that did not find an inverse association used 25(OH)D values from more than a decade prior to COVID-19 and in the multivariable analysis used some confounding factors that affect 25(OH)D	Table 1 and Table 2
Temporality	Four retrospective studies found inverse correlations between serum 25(OH)D and incidence of COVID-19 or SARS-CoV-2 positivity	[34,36,37,38]
Biological gradient	The large observational study of SARS-CoV-2 positivity found a large decrease as serum 25(OH)D increased from <20 to 50 ng/mL	[38]
Plausibility	Mechanisms have been proposed to explain how vitamin D reduces risk of SARS-CoV-2 infection and COVID-19	Discussed in this review
Coherence with known facts	Serum 25(OH)D concentrations are inversely correlated with risk and outcome of many diseases, also supported by RCTs in several cases	[5,7,8,44,139]
Experiment	Two intervention studies provide weak experimental support. Many RCTs are either planned or in progress to evaluate the role of vitamin D supplementation on COVID-19 risk and outcomes [18]	[58,59]
Analogy	Vitamin D supplementation reduces risk of some acute respiratory tract infections	[8]
Account for confounding factors	Univariate or multivariate regression analyses with confounding factors	[29,31,36,37]
